# Interprofessional education and its impact on learners’ attitudes over time: a cross-lagged panel analysis

**DOI:** 10.1186/s12909-026-08844-1

**Published:** 2026-06-09

**Authors:** Jean Anthony Grand-Guillaume-Perrenoud, Isabelle Peytremann-Bridevaux, Ingrid Gilles, Eva Cignacco

**Affiliations:** 1 Department of Health Professions, Bern University of Applied Sciences, Bern, Switzerland; 2 Department of Epidemiology and Health Systems, Unisanté, University Center for Primary Care and Public Health & University of Lausanne, Lausanne, Switzerland

**Keywords:** Interprofessional collaborative practice, Healthcare professionals, Attitude development, Attitude spillover, Longitudinal study, Cross-lagged panel model

## Abstract

**Background:**

Interprofessional education (IPE) prepares healthcare professionals (HCP) for interprofessional collaborative practice (IPCP) by fostering knowledge, skills, values, and attitudes. Attitudes trigger approach or avoidance motivation, making engagement with IPCP more likely when HCPs hold positive attitudes. As research has struggled to consistently demonstrate a positive, long-term impact of IPE on healthcare students’ attitudes, we investigated attitude development longitudinally in three undergraduate cohorts studying to become dieticians, midwives, nurses, or physiotherapists at a university of applied sciences in Switzerland. Specifically, we examined (1) whether related interprofessional attitude dimensions influenced each other over time, (2) how stable these dimensions were, (3) the impact of student characteristics, and (4) whether attitudes improved across time.

**Methods:**

We conducted a longitudinal study using structural equation modeling (SEM) to estimate cross-lagged panel models (CLPM) based on two out of three dimensions of the German version of the Interprofessional Attitudes Scale (G-IPAS): Teamwork, Roles, Responsibilities (TRR) and Community-Centeredness (CC). Of 879 undergraduate students who began their studies between 2018 and 2021, 234 provided data at least twice and were included in the final analyses.

**Results:**

No cross-lagged effects were found between TRR and CC, however they showed moderate to moderate-to-strong stability across time. Student characteristics influenced attitudes: semester of study was positively associated with CC, male sex was negatively associated with CC, dietetics students reported more positive CC attitudes, and physiotherapy students reported more negative TRR attitudes. TRR attitude scores decreased significantly over time, while a change in CC could not be verified due to inadequate model fit.

**Discussion:**

IPE did not produce the expected positive effect on interprofessional attitude development during undergraduate healthcare studies, in contrast to much previous research. The absence of reciprocal effects between attitude dimensions across time may indicate that they are serving different underlying psychological functions, with TRR being more utilitarian and CC being more value-oriented. Tailoring IPE curricula to the underlying psychological functions that interprofessional attitudes serve in different learner groups may enhance their effectiveness, thereby fostering positive and durable attitudinal change.

## Background

Healthcare systems rely on educational institutions to train the workforce required to provide their vital services [29]. These institutions, in turn, equip aspiring healthcare professionals (HCPs) with the knowledge, skills, and attitudes required to be prepared for practice in their chosen field [75]. With the current public health landscape in developed nations marked by an aging population and associated increases in chronic diseases, comorbidity, as well as surging healthcare costs, a more efficient use of healthcare resources is required [9, 13]. Interprofessional collaboration (IPC) is a vital tool to address the challenges, as it improves patient care and supports the reduction of healthcare costs [60, 73] by promoting egalitarianism among healthcare roles [68], shared responsibility and decision-making [11, 53], as well as facilitating the allocation of patient care to the HCP most appropriately trained for a given task [21].

IPC requires its own set of competencies, which can be categorized into requisite knowledge, skills, values, and attitudes [18, 38]. They are taught in undergraduate training in the form of interprofessional education (IPE), which is defined as the process of actively preparing aspiring HCPs for collaborative practice [18]. In this context, the frameworks developed by the Canadian Interprofessional Health Collaborative [17, 18] and the Interprofessional Education Collaborative [38] have the goal of guiding IPE curricular development and collaborative practice. The frameworks also describe some theoretical assumptions of how IPE is intended to work [31], represented for instance, in the conceptualization that knowledge, skills, values, and attitudes are the guiding foundations of practice behavior [17].

The idea that values and attitudes guide behavior has its roots in psychological theory. Values represent desirable goals and also serve as criteria of evaluation [65]. One of the values in the context of IPC is respectfulness [17], which can be articulated, for instance, as a goal (“HCPs should be respectful.”) or as evaluation criterion (“How respectful was the HCP?”). Attitudes, by comparison, are evaluations that are more narrowly circumscribed and relate to specific people, things, or ideas [6]. One can, for instance, have a positive attitude towards IPC or towards members of healthcare professions other than one’s own. Attitudes are particularly relevant for IPC because they activate approach or avoidance motivation [1, 22, 24], leading HCPs to engage more readily with collaborative situations or colleagues they view positively, and to withdraw from those they perceive negatively. The triggering of approach or avoidance motivation makes engagement with IPC more likely when HCPs hold positive attitudes [31, 42].

Most research shows that IPE improves attitudes towards IPC and HCPs whatever the profession [3, 4, 31, 70]. However, some research has also shown a decline in attitudes when IPE is experienced as difficult or of poor quality, when it causes boredom and frustration, or is perceived as unnecessary [3, 4, 67, 69]. A recent realist synthesis has identified several mechanisms through which IPE may foster positive attitudes, with one central mechanism being the development of trust, respect, and mutual liking among learners [31]. Despite these theoretical advances, research has struggled to consistently demonstrate a long-term positive impact of IPE on attitudes [66], with some studies successfully showing the maintenance of a positive attitude two months after attending IPE [44, 52], while studies looking at longer periods, such as 3 months [19], and 6 months [54] have failed to show any improvement after the time had elapsed. There is a dearth of research investigating attitude development over extended periods of time, with much research limited to a pre-IPE/post-IPE design and evaluating the effect of IPE at the end of a course (e.g., [5, 46, 72]). Some research extends follow-up to up to 6 months post-IPE (e.g., [19, 54, 76]). However, longitudinal studies evaluating interprofessional attitude development across several academic years appear especially scarce (e.g., [27, 43]).

While existing evidence suggests that IPE can foster interprofessional attitude development, often in a positive and sometimes sustained manner, important theoretical questions remain insufficiently explored. Particularly neglected are questions about how closely related attitudes influence one another [23, 34] and how stable interprofessional attitudes are over time [41]. Our objective is therefore to investigate interprofessional attitude development across up to three academic years among undergraduate healthcare students studying to become dietitians, midwives, nurses, or physiotherapists at a university of applied sciences in Switzerland. We address previous research shortcomings by testing how substantively related, adjacent interprofessional attitude dimensions may influence each other across up to three academic years. In addition, we tested how stable interprofessional attitude dimensions were, as this may indicate the degree to which they can be influenced through IPE. Finally, we investigated the influence of student characteristics, including age, sex, aspired profession, and study semester, as they have previously been shown to impact attitude outcomes [27, 43]. Guided by the theoretical reasons elaborated, we tested the following hypotheses: H1: Interprofessional attitude dimensions affect each other across time. H2: There is moderate stability within an attitude dimension across time. H3: Semester of study, age, sex, and aspired profession affect attitude outcomes. H4: There is a positive change in attitudes across time.

## Methods

We conducted a longitudinal study using structural equation modeling (SEM) to estimate cross-lagged panel models (CLPM) and latent mean differences.

### Study context and data collection

We collected the data of four undergraduate student cohorts studying to become dieticians, midwives, nurses, or physiotherapists, who began their studies in 2018, 2019, 2020, or 2021, respectively, at a university of applied sciences in the German-speaking region of Switzerland. These healthcare students attended IPE classes which were organized into modules and taught throughout all three-years of Bachelor studies. After completing each IPE module, students received an e-mail containing a link to an online survey. This survey collected students’ evaluations of the module they had just attended as well as their attitudes towards IPC.

To anonymously track each student across multiple surveys, a personal code was generated using responses to several non-identifiable questions. These included, for example, the first and last letter of the father’s and mother’s first name, the day of the month of their birth, and the year of birth. Each student could potentially take part in up to six surveys, two for each academic year.

We pooled the survey data from three academic years of the four study cohorts resulting in an initial sample of *N =* 879 students. Our original aim was to analyze data from students who participated in at least one survey for each academic year. However, only 37 students met this criterion. We therefore decided to include students who had participated in at least two survey waves, resulting in a final sample size of *N =* 234 students from the 2018, 2020, and 2021 cohorts. For each student we selected the earliest and the latest available survey data, which we labeled as “earlier wave” (t1) or “later wave” (t2) for longitudinal analysis. The data from these students were drawn from one of the following combinations, based on their survey completion: 1) the first and second semester, 2) the second and third semester, or 3) the first and third year of study.

### Survey instrument

The German version of the Interprofessional Attitudes Scale (G-IPAS) was used [58]. Adapted from the original 5 dimension scale [57], G-IPAS comprises three dimensions: 1) Teamwork, Roles, Responsibilities (TRR), 2) Patient-Centeredness (PC), and 3) Community-Centeredness (CC). Due to the longitudinal design of the study and the requirement for measurement invariance, only the TRR and CC dimensions were included in the analysis, the Patient-Centeredness dimension being omitted due to lack of measurement invariance. Additional variables collected included age (continuous), sex (male, female), profession (dietics, midwifery, nursing, physiotherapy), enrollment cohort (2018, 2019, 2020, 2021) and study semester (1st to 6th).

### Analysis

We conducted confirmatory factor analyses (CFA) with the TRR and CC dimensions of G-IPAS for each survey wave separately. The objective was to establish a measurement model using the same set of items for the two survey waves. The models were required to demonstrate acceptable fit in both waves and to achieve at least partial scalar invariance, in order to permit latent mean comparisons.

We refined the measurement models based on processes recommended by Byrne [10]. Based on model fit indices, theoretical considerations (e.g., item content domain redundancy, dimensionality), high residuals, and highly correlated errors, items were evaluated individually in iterations and removed if necessary. Error covariances were modeled based on conceptual similarity of their items and when modification indices suggested a significant shared variance existed that was not explained by the latent variable. We made our final item selection based on measurement validity and structural equivalence across waves, and used the factor structures that provided adequate fit and structural equivalence to test for measurement invariance. To assess configural, metric, scalar, and strict invariance models, we evaluated model fit indices and observed changes in CFI and RMSEA, wherein the changes should be ΔCFI ≤—0.01 and ΔRMSEA ≤ 0.015, respectively [14, 15].

The structural model was based on the basic structure of a cross-lagged panel model, where two variables are measured on at least two different time points and modeled to have a stability path for each variable across time, as well as cross-lagged paths from each variable at t1 to the other variable at t2. Control variables were modeled with an indicator variable to each of the two outcome latent variables TRR and CC, respectively. Non-significant control variable paths were removed iteratively. If a control variable had no significant path to either outcome latent variable, it was omitted from the model. We conducted a latent mean comparison between waves using multigroup analysis, with the latent construct of the earlier wave and later wave of each construct categorized as a group, fixing the latent mean to 0 at t1 and allowing the latent variable at t2 to be freely estimated. We set the variances of the latent variables to 1 in both waves to standardize the estimated parameter.

Item non-response ranged from zero to three missing responses (1.3% overall). Missing data were handled using full information maximum likelihood (FIML) [45]. All models were estimated using maximum likelihood, with bootstraps using 1000 samples applied to derive robust standard errors and confidence intervals. Model goodness of fit was assessed using absolute (RMSEA, SRMR) and comparative fit indices (CFI, TLI) [37]. Proposed cutoffs were applied to establish adequacy of fit. These are: RMSEA < 0.08.

 [8], SRMR < 0.08, CFI and TLI > 0.90 [10]. Discriminant validity was assessed using the Fornell-Larcker criterion [28], wherein each construct’s average variance extracted is compared to its correlation with the other construct and, when higher, is interpreted to indicate adequate discriminant validity.

We conducted descriptive analyses in SPSS Statistics v. 29 and structural equation modeling in SPSS Amos v. 29.

## Results

### Respondents’ characteristics

The students were an average of 22.6 years old during the earlier wave (SD = 4.1) and an average of 24.4 years of age in the second wave. Female students accounted for 201 participants in the sample (85.9%), while males made up 33 participants (14.1%). Physiotherapy students were the largest professional category with 88 participants (37.6%). Students from the 2020 enrollment cohort were the largest group with 102 participants (43.6%). The majority of students (182 participants, 77.8%) were in their first semester in the earlier study wave. The majority of students (159 participants, 67.9%) were in their sixth and final semester in the later study wave (Table 1).

**Table 1:** Participant characteristics

		Earlier wave ***N =*** 234	Later wave ***N =*** 234	Total ***N =*** 234	Percent
Age		M = 22.6 SD = 4.1	M = 24.4 SD = 4.1	-	
Sex	Male	-	-	33	14.1
	Female	-	-	201	85.9
Aspired Profession	Dietetics	-	-	50	21.4
	Midwifery	-	-	42	17.9
	Nursing	-	-	54	23.1
	Physiotherapy	-	-	88	37.6
Cohort	2018	-	-	48	20.5
	2020	-	-	102	43.6
	2021	-	-	84	35.9
Semester	First	182	-	-	77.8
	Second	-	-	-	
	Third	4	69	-	1.7 (29.5)^a^
	Fourth	5	6	-	2.1 (2.6)^a^
	Fifth	43	-	-	18.4
	Sixth	-	159	-	67.9

^a^Figures in parenthesis relate to the later wave

### Confirmatory factor analyses

The final measurement model for TRR was composed of 6 items out of 9 (Table 2). The final model for CC was composed of 5 items out of 7. Both models demonstrated high composite reliabilities in both waves (≥ 0.85) (Table 2). All standardized factor loadings were significant (*p <* 0.001). We measured indicator reliabilities from 0.27 to 0.76, with the lowest reliability observed in TRR Item 8 (“Not necessary to learn together”) at Wave t1. We computed an average variance extracted between 0.48 and 0.61. This met the minimum threshold of 0.40 in both measurement models and indicated adequate convergent validity. The measurement models showed adequate to good fit across the two waves in the configural model (RMSEA = 0.047 and 0.064, CFI > 0.97) (Table 4). The measurement models demonstrated adequate discriminant validity as indicated by the Fornell-Larcker criterion (Table 3). While full scalar invariance was not possible for TRR, we were able to achieve partial scalar invariance (Table 4) by freeing the intercept of Item 8 (“Not necessary to learn together”). Strict measurement invariance could not be established for TRR (ΔCFI =—0.017), but was achievable for CC.

**Table 2:** Confirmatory factor analyses and measures of fit

Dimension	Item	Mean	SD	Indicator Reliability	t-Value of Factor Loading	Cronbach’s α	Composite Reliability	Average Variance Extracted
Teamwork, Roles, Responsibilities		-	-	-	-	.86 (.85)	.87 (.85)	.52 (.48)
	trr1: Shared learning helps to become better team worker	3.6 (3.1)	1.0 (1.1)	.61 (.59)	^a^			
	trr3: Learning with others helps to become effective	3.7 (3.2)	1.0 (1.1)	.61 (.57)	12.16*** (11.00***)			
	trr4: Shared learning increases ability to understand	3.7 (3.3)	.9 (1.0)	.52 (.40)	10.89*** (9.19***)			
	trr6: Shared learning helps communicate better	3.8 (3.4)	1.0 (1.1)	.56 (.60)	11.69*** (11.39***)			
	trr7: I welcome opportunity to work on group projects	3.1 (2.9)	1.1 (1.2)	.50 (.42)	10.60*** (9.26***)			
	trr8: Not necessary to learn together	2.3 (2.3)	1.1 (1.1)	.27 (.30)	8.57*** (7.90***)			
Community-Centeredness		-	-	-	-	.85 (.90)	.85 (.89)	.53 (.61)
	cc1: Important to work with public health administrators, policy makers	4.3 (3.1)	.70 (1.1)	.48 (.55)	^a^			
	cc2: Important to work to promote community and public health	4.3 (3.5)	.81 (1.0)	.59 (.73)	9.98*** (12.66***)			
	cc3: Important to work with legislators to develop laws, policies	4.2 (3.2)	.80 (1.1)	.70 (.76)	10.50*** (12.84***)			
	cc4: Important to work with nonclinicians	4.2 (3.3)	.86 (1.0)	.50 (.50)	8.30*** (11.87***)			
	cc5: Important to focus on populations, communities and individuals	4.2 (4.3)	.81 (.79)	.41 (.52)	8.47*** (10.62***)			

The first figure of a cell pertains to Wave t1, figures for Wave t2 are reported in parenthesis

^a^Parameter set to value of 1 for model identification

^***^p < 0.001

**Table 3:** Test of discriminant validity

Nr	Factor	1	2
1	Teamwork, Roles, Responsibilities	**0.72**^a^(0.69)^1^	0.31*** (0.32***)^1^
2	Community Centeredness	0.31*** (0.32***)^1^	**0.73**^a^(0.78)^1^

^1^Figures in parentheses computed from t_2_ scores

^a^Square root of the average variance extracted of a factor. The Fornell-Larcker Criterion of discriminant validity is satisfied when a factor’s square root of average variance extracted exceeds the value of its correlation with the other factors

^***^p < 0.001

**Table 4:** Measurement invariance across Waves t1 and t2

Dimension	Invariance model	χ^2^ (df)	CFI	RMSEA	Δ CFI	Δ RMSEA
Teamwork, Roles, Responsibilities (TRR)	Configural	40.60 (14)	0.976	0.064	-	-
	Metric	43.72 (19)	0.978	0.053	+ 0.002	−0.011
	Partial Scalar	55.15 (23)	0.971	0.055	−0.007	+ 0.002
	Strict	80.30 (29)	0.954	0.062	−0.017	+ 0.007
Community-Centeredness (CC)	Configural	12.05 (6)	0.995	0.047	-	-
	Metric	16.68 (10)	0.994	0.038	−0.001	−0.009
	Scalar	20.00 (14)	0.995	0.030	+ 0.001	−0.008
	Strict	28.57 (19)	0.991	0.033	−0.004	+ 0.003

### Cross-Lagged Panel Models (CLPMs)

We constructed four CLPMs, each corresponding to a separate hypothesis.

#### Hypotheses 1 and 2: cross-lagged panel model

**Fig. 1:**
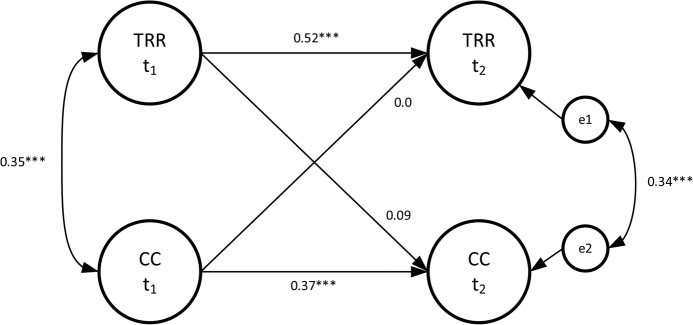
Cross-lagged panel model of two IPAS dimensions Note: *** *p <* 0.001

We hypothesized that the two different dimensions of interprofessional attitudes, TRR and CC, affect each other across time (Fig. 1). The cross-lagged path TRR(t1) → CC(t2) was not significant at $\beta =$ 0.00 (CI [−0.06, 0.24]) (Table 5). The second cross-lagged path CC(t1) → TRR(t2) was also not significant ($\beta =$ 0.09; CI [−0.15, 0.16]). The stability path TRR(t1) → TRR(t2) (0.52; CI [0.38, 0.64]; *p <* 0.001) was significant and showed moderate to strong stability. The second stability path CC(t1) → CC(t2) (0.37; CI [0.22, 0.51]; *p <* 0.001) was also significant, still showed moderate stability but with a lower path coefficient. The model accounted for 27% of the variance in the TRR(t2) latent variable and 17% of the variance in the CC(t2) latent variable. Fit indices indicated a good model fit. The hypothesis that TRR and CC affect each other across time must be rejected due to the non-significant path coefficients.

**Table 5:** Cross-lagged panel model effects

Model	Path	Beta	95% CI
CLPM (Fig. 1)^1^			
RMSEA = 0.053; CFI = 0.948; TLI = 0.938; SRMR = 0.054	TRR (t1) → CC (t2)	0.00	−0.06—0.24
	CC (t1) → TRR (t2)	0.09	−0.15—0.16
	TRR (t1) → TRR (t2)	0.52***	0.38—0.64
	CC (t1) → CC (t2)	0.37***	0.22—0.51
CLPM controlling for semester, sex, profession (Fig. 2)^2^			
RMSEA = 0.058; CFI = 0.914; TLI = 0.902; SRMR = 0.070	TRR (t1) → CC (t1)	0.01	−0.04—0.26
	CC (t1) → TRR (t2)	0.11	−0.15—0.16
	TRR (t1) → TRR (t2)	0.50***	0.37—0.64
	CC (t1) → CC (t2)	0.34***	0.18—0.47
	Semester → CC (t2)	0.18**	0.05—0.29
	Male sex → CC (t2)	−0.16**	−0.28—−0.05
	Dietetics → CC (t2)	0.18**	0.05—0.30
	Physiotherapy → TRR (t2)	−0.17**	−0.30—−0.05

^**^p < 0.01, ***p < 0.001

#### Hypothesis 3: cross-lagged panel model controlling for semester, age, sex, and aspired profession

**Fig. 2:**
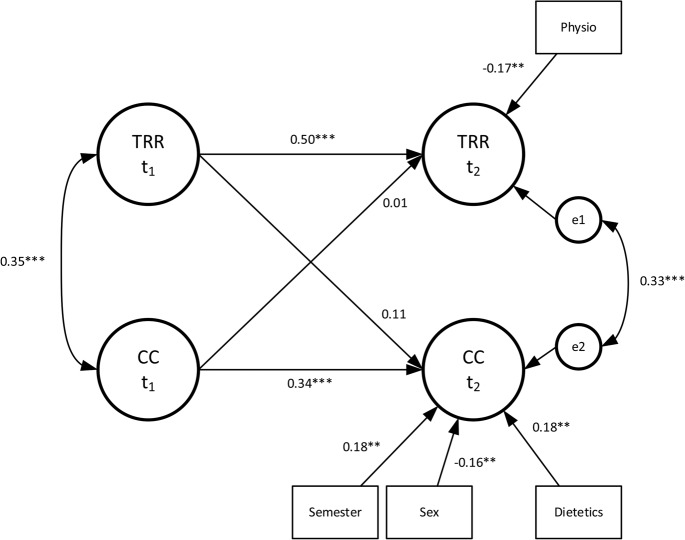
Cross-lagged panel model controlling for semester, sex, and profession. Note: ** *p <* 0.01, *** *p <* 0.001

We hypothesized that the semester of study and the student’s sex affect attitude outcomes (Fig. 2). This model expands the previous model with the covariates semester of study, age, sex, and aspired profession. The initial modeling included age as a control variable, but was later omitted, due to non-significance. The estimates for the cross-lagged paths remained robust but are slightly higher compared to the previous model. They remained non-significant ($\beta =$0.01; CI [−0.04, 0.26]) and ($\beta =$0.11; CI [−0.15, 0.16]) (Table 5). The stability paths remained robust but were slightly lower, with TRR(t1) → TRR(t2) at $\beta =$0.50 (CI [0.37, 0.64]; *p <* 0.001) and CC(t1) → CC(t2) at $\beta =$0.34 (CI [0.18, 0.47]; *p <* 0.001). This model explained 29% of the variance in the TRR(t2) latent variable and 24% of the variance in the CC(t2) latent variable, explaining an additional 2% of explained variance in the TRR latent variable and an additional 7% in the CC latent variable. Model fit was poorer but was acceptable. The model showed a positive effect of being in a higher semester on CC ($\beta =$0.18; CI [0.05, 0.29]; *p <* 0.01) and a negative effect of male sex on CC ($\beta =$−0.16; CI [−0.28, −0.05]; *p <* 0.01). In addition, two profession covariates showed significant associations. Aspiring to become a dietician had a positive impact on CC ($\beta =$0.18; CI [0.05, 0.30]; *p <* 0.01). While aspiring to become a physiotherapist had a negative impact on TRR ($\beta =$−0.17; CI [−0.30, −0.05]; *p <* 0.01). The hypothesis that semester of study, age, sex, and aspired profession affect attitude outcomes can be partially accepted. Age had no significant impact on either outcome variable, while semester and sex affected the CC dimension but not the TRR dimension. The hypothesis that the aspired profession affects attitude outcomes can be accepted in the case of dietetics and physiotherapy.

#### Hypothesis 4: positive change in attitudes across time

We hypothesized that the attitude dimensions measured would show a positive change across time. Under partial scalar invariance we found a significant attitude decrease in the TRR dimension in the later wave, compared to the earlier wave (Estimate = −0.445, SE = 0.101, CR = − 4.383, *p <* 0.001). Model fit was adequate (RMSEA = 0.068; CFI = 0.954; TLI = 0.942; SRMR = 0.066).

After establishing scalar invariance in the CC dimension and setting model constraints to estimate the t2 mean, we found that the constraints deteriorated model fit substantially below established cutoffs (RMSEA = 0.376; CFI = 0.179; TLI = −0.173; SRMR = 0.191). This prevented the latent mean estimate for the CC dimension from being interpreted. The hypothesis that there is a positive change in attitudes across time must be rejected for the TRR dimension, because the attitude becomes poorer. The hypothesis could not be verified for the CC dimension due to inadequate model fit.

## Discussion

This study investigated the longitudinal development of interprofessional attitudes among undergraduate healthcare students across several healthcare professions. The main findings show that IPE did not have the intended positive impact on interprofessional attitude development during undergraduate healthcare studies. Indeed, we found the Teamwork, Roles, Responsibilities (TRR) attitude dimension to have decreased significantly, while a change in Community-Centeredness (CC) could not be statistically verified. In addition, the TRR and CC attitude dimensions did not influence each other across time, contrary to theoretical expectation. However, they were moderately to strongly stable temporally, suggesting a degree of attitude malleability. What we discovered to have a significant impact on the individual development of interprofessional attitudes were learner characteristics, such as semester of study, sex, and aspired profession.

We expected the two attitude dimensions TRR and CC to mutually affect each other, as the literature suggests that attitudes in one domain can affect, i.e., spill over, into another, when domains are intertwined or interrelated [63]. Attitude spillover has, for example, been proposed between work satisfaction and life satisfaction [62, 63]. Likewise, a positive attitude in one interprofessional attitude dimension can potentially influence another interprofessional attitude dimension. One underlying mechanism for attitude spillover is the psychological need for consistency, a lack of which causes internal tension and emotional discomfort known as cognitive dissonance [16, 26, 33, 35]. A related explanation for attitude spillover is that similar attitudes are part of associated semantic networks that tend to cognitively activate together due to their relatedness [24, 25].

The fact that we did not find a cross-lagged effect may indicate that the attitude dimensions may be serving different underlying psychological needs, despite being conceptually related [39, 50]. The psychological needs that attitudes satisfy have also been described as their function [12, 50, 51]. Some attitudes may be described as serving a utilitarian function, while others may serve a value-expressive function [12, 20, 39, 48, 49]. The TRR attitude dimension can be described as having a stronger utilitarian function, because it is related to specific goals, such as becoming a better team worker and learning with other disciplines to become more effective. In comparison, the CC dimension may be described as being more value-expressive, as it allows HCPs to express cherished ideals [39, 50], e.g., the HCP’s commitment to work to support the community and public health. Because TRR and CC may be associated with different underlying psychological functions, they may activate together less frequently than one would expect from their conceptual similarity, thereby reducing contemporaneous semantic network activation [24, 25]. Indeed, it has been suggested that associative connections between attitudes are not always founded on semantic relatedness, but may instead reflect an HCP’s idiosyncratic learning history [50]. These findings provide support for the multidimensional and potentially developmentally independent nature of interprofessional attitudes (cf. [43, 57]).

Our finding of moderate to moderate-to-strong stability of interprofessional attitudes indicates a degree of malleability by external factors, which could be strategically utilized in IPE. Attitudes are generally considered relatively stable cognitive structures [6, 36, 47]. As such, stability is one of the factors that make up an attitude’s strength. When an attitude is strong, it encompasses that it is stable, meaning it does not tend to change over time, it is resistant to influence, and has a tendency to affect a person’s thinking and behavior [36, 64]. Another factor that is an element of attitude strength is its importance, which relates to an HCP’s subjective judgment of an attitude’s significance. Importance is linked to three vital factors: 1) an HCP’s self-interest, 2) their identification with personally significant groups or individuals, and 3) their values [7, 20, 36].

Developing attitudes that are strong should be a focus of IPE, as strong attitudes are the ones most likely to shape practice behavior [20, 36]. IPE can develop stronger and more behaviorally consequential interprofessional attitudes by targeting the factors that are linked with attitude importance. This more persuasive IPE can be developed by tailoring IPE messaging to fit an underlying attitude function [40]. For instance, highlighting how IPE is career-relevant and helps HCPs to deal with day-to-day issues in healthcare can improve attitudes towards IPE [61], perhaps by demonstrating that IPCP also contributes to an HCP’s self-interest. When IPE fosters good relationships among learners [3, 4, 55, 69], learners regard their peers as an important reference group [2], which can help to mutually reinforce each other’s appreciation for IPCP [56]. Finally, IPE can help strengthen attitudes by allowing them to be internalized and become part of the HCP’s own value system [20, 31, 71].

The lack of positive attitude development that we observed longitudinally in our study diverges from the overall trend of attitude improvement post-IPE in the literature [31, 32, 66]. This may be due to our longer observation period compared to the shorter-term pre-/post-intervention studies that make up the bulk of IPE effectiveness research. However, lack of significant positive attitude development or even a negative development among students with certain characteristics have been previously noted. For instance, [3, 4] have found a significant decline in attitudes among medical students in the clinical, compared to the pre-clinical years. Similarly, Bloomfield et al. [5] found that IPE did not significantly change attitudes among medical students. Other studies found physicians to have lower attitude scores than other healthcare professionals, both before and after IPE [54, 76]. The link between negative attitudes and profession may be associated with the status and decision-making authority afforded to certain professions, especially the medical profession, which they may be reluctant to share [74]. A fear of status loss has in fact already been observed in students [3, 4, 30]. This may explain why being a physiotherapy student was associated with more negative TRR attitudes in our study. As we found in our own study, male sex was also associated with poorer attitudes in several other studies [3, 4, 27, 76]. This has been attributed to the communication style in IPE which may better suit women’s preference for relationship-building [27]. Finally, while we found a positive association in our study, previous evidence is mixed regarding the association of semester or year of study to having more positive interprofessional attitudes, ranging from no association [43], to a negative association [3, 4, 59], and a positive association [27]. Overall, our findings underscore the vital role of learner-specific characteristics in shaping interprofessional attitudes [31].

### Strengths and limitations

This study features several methodological and conceptual strengths. Indeed, it is among few longitudinal investigations of interprofessional attitude development in healthcare education. Using a cross-lagged panel model, we accounted for attitude stability over time, which provided more accurate estimates of attitudinal change and reduced inflated correlations. Also, we employed a stepwise, theory-driven modeling strategy, which produced robust estimates across all models, and the study drew on established psychological frameworks and concepts rarely applied to IPE, including functional attitude theory and attitude spillover, which offer new insights into the dynamics of interprofessional attitude development. Finally, having collected data from multiple healthcare professions and enrollment cohorts using the widely adopted IPAS instrument, our results offer comparability with other IPE studies.

This study’s limitations include that data were drawn from a single institution, which may limit generalizability. Also, although widely used, the IPAS instrument has known issues concerning ceiling effects and factor structure stability. Additionally, while our initial data set was large, our final data set included data from only 234 students who provided data on at least two time points, introducing potential non-response bias. Finally, by pooling data across varying time intervals, we could not meet the assumption of temporal consistency, limiting a more fine-grained analysis of developmental patterns.

## Conclusion

In contrast to much of the IPE literature, we found no evidence of sustained positive attitude development, nor of spillover effects between interprofessional attitude dimensions. Instead, our findings point to the relative stability of interprofessional attitudes and the importance of learner characteristics such as sex, profession, and semester of study. These results suggest that IPE interventions should move beyond a one-size-fits-all approach. Tailoring curricula to the distinct functions that attitudes serve and to the characteristics of specific learner groups may enhance their persuasiveness and improve the likelihood of producing durable, practice-relevant attitudinal change.

## Data Availability

Data sets analysed in the current study are available from the corresponding author on reasonable request.
